# Functional genetic signatures of the gut microbiome in cardiometabolic diseases: mechanisms and translational opportunities

**DOI:** 10.3389/frmbi.2026.1847345

**Published:** 2026-07-24

**Authors:** Martin Nganga Muigano

**Affiliations:** 1 Bio One Scientific, Nairobi, Kenya; 2 Institute of Biotechnology Research, Jomo Kenyatta University of Agriculture and Technology, Nairobi, Kenya

**Keywords:** bile acid metabolism, CAZymes, functional metagenomics, genetic signatures, gut microbiota, metagenomics, short-chain fatty acids (SCFAs), TMAO

## Abstract

The human gut microbiome plays a very important role in the regulation of host metabolism and overall physiological homeostasis. Disruptions in microbial community function have been increasingly implicated in cardiometabolic diseases, including obesity, type 2 diabetes, cardiovascular disease, and metabolic dysfunction-associated liver disease. Advances in metagenomic sequencing have identified functional genetic signatures within the gut microbiome for short-chain fatty acid biosynthesis, bile acid metabolism, lipopolysaccharide (LPS) production, amino acid metabolism, trimethylamine N-oxide (TMAO) generation, and carbohydrate-active enzymes (CAZymes). Across cardiometabolic conditions, a consistent pattern emerges of depletion of beneficial metabolic functions and enrichment of pro-inflammatory and metabolically disruptive pathways. These findings point to the importance of microbial functional capacity, rather than taxonomic composition alone, in shaping disease risk and progression. This review explores the functional genetic signatures for cardiometabolic diseases and translational potential of these signatures including their potential roles as diagnostic biomarkers, therapeutic targets, and tools for precision therapy. This understanding of microbiome-derived functional pathways may inform the development of targeted strategies aimed at restoring metabolic balance and improving cardiometabolic health.

## 1 Introduction

The human gut microbiome comprises a complex and metabolically versatile community of microorganisms that has co-evolved with its host over millennia into a symbiotic relationship that is essential for health and well-being (Rook et al., 2017; Muigano et al., 2025). This ecosystem encompasses bacteria, archaea, viruses, and fungi that collectively encode various genes of diverse functional diversity. The rich microbial pool contributes to digestion, nutrient synthesis, immune system maturation, and the regulation of host metabolism (Brestoff and Artis, 2013; Rooks and Garrett, 2016; Postler and Ghosh, 2017). This integrated host-microbial system has led to some researchers calling the human being a “superorganism” whose microbial partners contribute substantially to the overall physiological homeostasis (Salvucci, 2019; Russo et al., 2024). This superorganism carries trillions of diverse microbial cells particularly in the gut environment (Gentile and Weir, 2018). Advances in culture-independent techniques like high-throughput sequencing and metagenomic analyses have provided unprecedented resolution of the gut microbiome. These endeavors have enabled researchers to identify not only the taxonomic composition but also the genetic and functional profiles of resident microbes (Tanca et al., 2017; Chu et al., 2021; Mancabelli et al., 2024). These studies have revealed that specific microbial genes and pathways, here collectively referred to as genetic signatures, are associated with both health and disease. For example, genes associated with production of short-chain fatty acids (SFAs), bile acid metabolism, and xenobiotic degradation are consistently enriched in healthy individuals (Koppel et al., 2018; Clarke et al., 2019; Ramos Meyers et al., 2022). To the contrary, adversarial shifts in microbial genetic repertoires are often observed in metabolic, inflammatory, and neurodegenerative disorders (Chen et al., 2025; Li and Lu, 2025). Such genetic signatures offer a framework for understanding how microbiota contribute to disease pathogenesis, as well as for developing predictive and personalized interventions.

Disruptions in microbial composition, often termed dysbiosis, have been implicated in a wide range of disease states, including obesity, type 2 diabetes, inflammatory bowel disease, cardiovascular disease, and neuropsychiatric conditions (Dubinski et al., 2021; Vamanu and Rai, 2021; Shabani et al., 2025). Beyond disease risk, the gut microbiome influences drug metabolism, efficacy, and toxicity, highlighting its role as a determinant of inter-individual variability in treatment response (Thursby and Juge, 2017). Consequently, identifying microbial genetic markers with predictive or diagnostic value has become a central goal in microbiome research, with significant implications for precision medicine. Yet, the composition and function of the gut microbiome are highly individualized as a result of host genetics, diet, environment, and early-life microbial exposure (Thursby and Juge, 2017; Johnson, 2020). Despite this variability across individuals, a set of core bacterial phyla like Firmicutes, Bacteroidetes, Actinobacteria, and Proteobacteria are predominant across healthy adults where they contribute to essential physiological processes such as immune modulation, epithelial integrity, and energy homeostasis (Rinninella et al., 2019; Hou et al., 2022). Recent studies point to the likelihood that strain-level differences and the presence or absence of specific functional genes can have significant influence on host physiology (Patnode et al., 2021; Lynch et al., 2023). This suggests that health outcomes are shaped not only by the presence or absence of certain species but also by their precise genetic repertoire. The recognition has shifted the focus from taxonomic profiling to functional genomics, which emphasizes the relevance of microbial gene content in mediating host health and disease.

Existing reviews have advanced our understanding of gut microbial metabolites and functional metagenomics in human disease. For example, a review by Fan and Pedersen consolidated the research on the role of human gut microbiota in metabolic health and disease (Fan and Pedersen, 2021). Another review synthesized the research on microbe-derived metabolites and their role in human health (Brown and Hazen, 2017). Elsewhere, Singh and colleagues reviewed the role of gut microbiota on the pathogenesis of metabolic syndrome (Singh et al., 2025) while a recent review focused on (Xie et al., 2026)youthful populations. These reviews tend to focus on microbial taxonomy, metabolite biology, or disease-specific mechanisms. What is comparatively underdeveloped is a cardiometabolic disease-focused synthesis that is simultaneously gene/pathway-centric and translational-focused. This review is positioned to address that gap. In light of the rapidly expanding literature on microbial functional genes, there is a need for a review from a gene-centric perspective. This review brings together the findings of shared and disease-specific functional signatures across cardiometabolic disorders. It aims to provide an integrated discussion of their biological and translational significance.

In this review, I synthesize current knowledge on the genetic signatures of the gut microbiome in cardiometabolic diseases. I highlight their mechanistic roles, gene function, and potential translational applications. I also discuss the future directions necessary for integrating microbiome genetics into precision and translational medicine. By examining the intersection of microbial genomics and host physiology, this review aims to provide a comprehensive perspective on the potential of the gut microbiome to inform personalized healthcare strategies.

## 2 Methodology

This study adopted a narrative review methodology. An extensive search was conducted on major scientific databases like PubMed, Google Scholar, and Web of Science. The search was based on a combination of key terms including “gut microbiome,” “functional genetic signatures,” “metagenomics,” “short-chain fatty acids,” “bile acid metabolism,” “lipopolysaccharide,” “TMAO,” “CAZymes,” “metabolic disease,” and “cardiometabolic disease.” The searches were supplemented by iterative snowballing from the reference lists of key articles. Searches were restricted to peer-reviewed, English-language publications. A two-step screening method was employed where article titles and abstracts were first screened followed by full texts review. Only peer reviewed articles with relevance to the study were included. Where multiple studies addressed the same gene-disease association, priority was given to larger cohort studies, mechanistic validation work, and meta-analyses where available.

## 3 Core functional genetic signatures in a healthy gut

**Figure 1:**
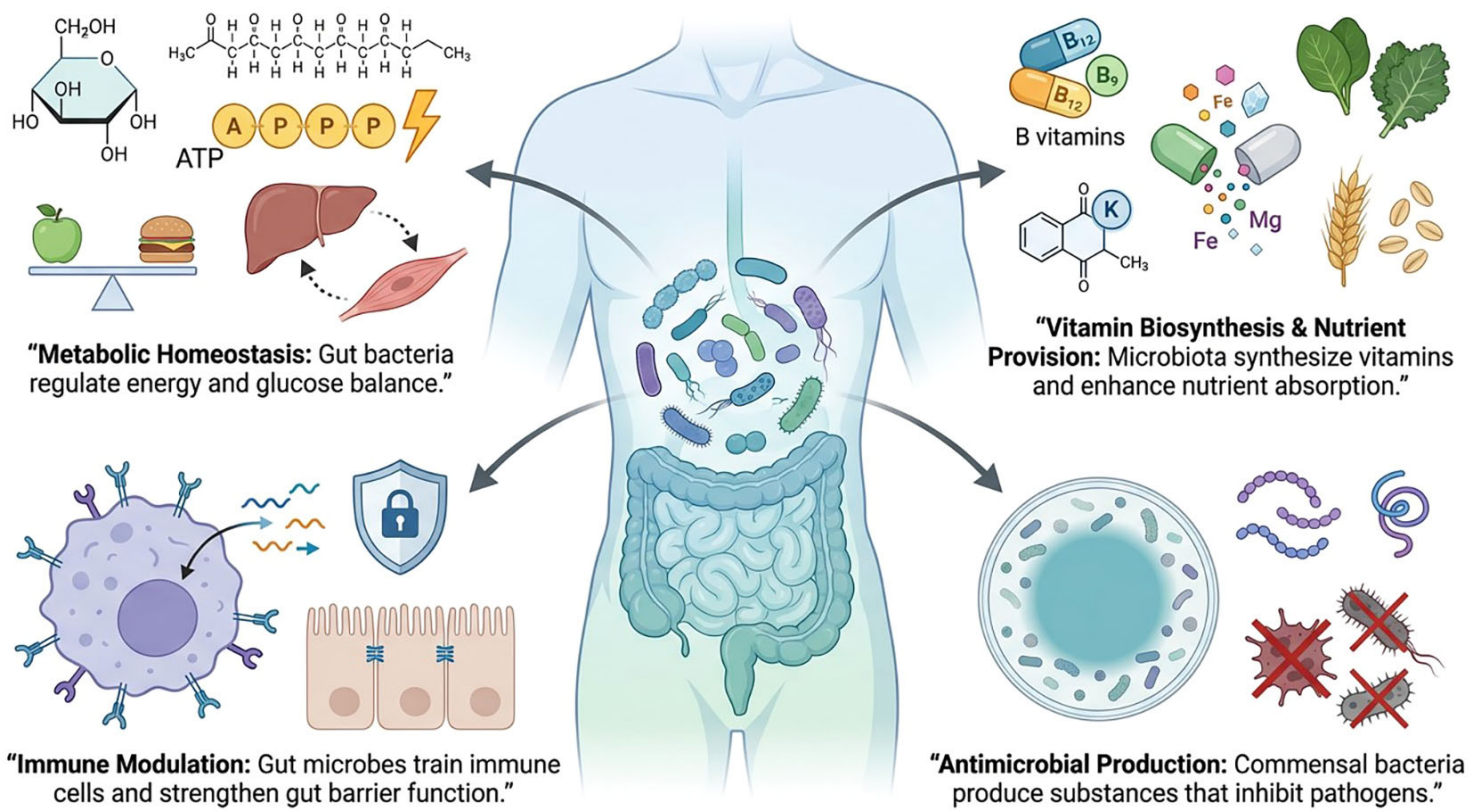
Functional roles of gut microbiome genetic signatures in maintaining host health. The gut microbiota encodes a diverse repertoire of genes that support multiple physiological processes essential for host well-being. These microbial functions include regulation of metabolic homeostasis and energy balance, biosynthesis of essential vitamins and nutrients, modulation of immune responses, and production of antimicrobial compounds that protect against pathogenic organisms. These genetic and metabolic activities highlight the central role of the gut microbiome in maintaining intestinal and systemic health.

The gut microbiome contributes to human health by supporting host physiology and metabolic stability. In these roles, a range of genes and pathways are involved. These genetic capabilities enable the microbiota to perform key roles in nutrient metabolism, vitamin production, immune regulation, and protection against pathogens. Rather than acting as passive inhabitants, gut microbes function as an active metabolic and immunological partner to the host. As illustrated in **Figure 1**, microbial genes and metabolic pathways collectively support several core processes that maintain physiological balance. These include regulation of energy metabolism, biosynthesis of essential vitamins and nutrients, modulation of immune responses, and production of antimicrobial compounds that limit pathogen colonization.

One of the essential roles of the gut microbiome is that it contributes to the regulation of host energy metabolism. It acts as a metabolic organ owing to the fact that it is equipped with a vast enzymatic arsenal that is capable of extracting calories from otherwise indigestible substrates and transforming them into metabolites beneficial to the host. Metagenomic analyses have confirmed that the healthy gut microbiome carries a rich repertoire of genes involved in carbohydrate degradation and fermentation. These include polysaccharide utilization loci (PULs), glycoside hydrolases (GHs), and carbohydrate-binding modules (CBMs), which together facilitate the breakdown of dietary fibers, resistant starches, and host-derived glycans (Flint et al., 2008; Puhlmann and de Vos, 2022; Crouch et al., 2024).

The fermentation of carbohydrates culminates in the production of short-chain fatty acids (SCFAs) namely acetate, propionate, and butyrate. In the gut, SCFAs are produced through the fermentation of fibers for purposes of contributing to signaling molecules, immune modulation, and tissue-specific energy production (Shin et al., 2023). This happens through gene-encoded enzymatic pathways that are particularly enriched in members of the Firmicutes phylum. Genes such as *but* (butyryl-CoA-transferase), *bcd* (butyryl-CoA dehydrogenase), *buk* (butyrate kinase), and *pta*/*ack* (phosphate acetyltransferase/acetate kinase) are key markers of this metabolic capacity (Shin et al., 2023).

The gut microbiome also serves an important role in the biosynthesis and supply of essential vitamins and cofactors that the host either lacks the capacity to synthesize or produces in insufficient amounts. Metagenomic surveys have consistently shown that genes encoding vitamin biosynthetic pathways are widely distributed among commensal taxa (Tarracchini et al., 2024; Yu et al., 2025).

Beyond the B vitamins, the gut microbiota contributes to the synthesis and salvage of other micronutrients, including vitamin K (menaquinones). These microbial menaquinones play vital roles in blood coagulation and bone metabolism, and their abundance has been inversely linked to inflammatory bowel disease and cardiovascular risk [14]. Dietary patterns and antibiotic exposure profoundly influence the abundance and expression of these vitamin biosynthetic genes.

The gut microbiota has evolved over time to offer the host protection against pathogens and maintenance of the immune system. This is evident from the fact that disruptions of gut microbial communities may be accompanied by immune dysregulation and the onset of autoimmune diseases (Wu and Wu, 2012). Gut microbes modulate the host immunity through a network of genetic pathways that regulate epithelial barrier integrity, immune tolerance, and inflammatory responses.

Barrier strengthening is also supported by microbial genes that regulate mucin degradation and host-glycan foraging. For example, gut Bacteroidetes encode sulfatases that remove sulfate groups from mucins, thereby releasing underlying O-glycans for further breakdown. Moreover, sialidases such as those encoded by *nanA, nanE, and nanK* are functionally active in *Faecalibacterium prausnitzii*, *Fusobacterium nucleatum*, *Lactobacillus sakei*, *Lactobacillus plantarum*, and *Lactobacillus salivarius* (Berkhout et al., 2022) while fucosidases (e.g., *AmGH29A, AmGH29B, AmGH29C, AmGH29D, AmGH95A* and *AmGH95B* loci) are functionally active in *Akkermansia muciniphila* (Shuoker et al., 2023) where they aid in the removal of sialic acid and fucose caps from mucin glycans. These activities of the enzymes encoded by these genes help in maintaining intestinal mucosal barrier, hence the overall immune and metabolic homeostasis (Shuoker et al., 2023).

Gut microbiota have also been linked to the production of a variety secondary metabolites with antimicrobial potential. Many of these compounds are encoded by biosynthetic gene clusters (BGCs) such as polyketide synthase (PKS) and nonribosomal peptide synthetase (NRPS) (Wang et al., 2019). Besides the BGCs, bacteriocin genes (e.g. class I and II types) have been identified in a broad range of gut commensals including the lactic acid bacteria (LAB) (Garcia-Gutierrez et al., 2018). Bacteriocins are ribosomally synthesized peptides with both broad- and narrow-spectrum antibiotic activities. For example, bacteriocins produced by *Ruminococcus gnavus* are active against *Bacteroides sp*, *Clostridium sp*, *Bifidobacterium sp*, and *Bacillus cereus* (Cui et al., 2012). Bacteriocins can inhibit the growth of competing bacteria as a strategy for limiting collateral damage to the commensal. Investigations by Zhang and colleagues has revealed the genetic signatures of commensal LAB by uncovering more communities 2,800 genes involved in antagonistic bacteriocins expression (Zhang et al., 2023).

## 4 Gut microbiome genetic signatures in cardiometabolic diseases

### 4.1 SCFA pathway depletion and metabolic dysfunction

Metabolic disorders occur when normal metabolic processes are altered due to factors like genetic predispositions and lifestyle choices. However, evidence in the past few decades has consistently established a link between these disorders and imbalances in gut microbiota. For example, gut dysbiosis has been associated with insulin resistance and non-alcoholic fatty liver disease (NAFLD) (Park et al., 2021). Among the most reproducible and mechanistically coherent functional signatures identified in metagenomic studies of cardiometabolic disease is the depletion of genes encoding the short-chain fatty acid (SCFA) biosynthetic machinery. This is due to the fact that gut microbiota-synthesized SCFAs may affect insulin sensitivity, intestinal barrier integrity, and systemic inflammation (He et al., 2020; Yao et al., 2022). Additionally, SCFAs act as key microbial metabolites that mediate communication between the gut microbiome and host metabolic systems. These molecules exert their effects primarily through receptor-mediated signaling pathways and epigenetic mechanisms, thereby influencing multiple aspects of cardiometabolic physiology (Lee et al., 2024). Butyrate, for example, modulates epithelial barrier integrity and inflammation via histone deacetylase inhibition and activation of G-protein–coupled receptors GPR41 and GPR43 (Salvi and Cowles, 2021). Propionate and acetate produced in Bacteroides and Veillonella serve as substrates for hepatic gluconeogenesis and lipid metabolism (Rios-Covian et al., 2017). These processes illustrate how microbial gene networks extend the metabolic scope of the host and maintain systemic energy equilibrium. Short-chain fatty acids exert diverse effects on host metabolism through receptor-mediated signaling and epigenetic regulation. The principal mechanisms through which these microbial metabolites influence cardiometabolic processes are summarized in **Table 1**.

**Table 1:** Mechanistic roles of short-chain fatty acids in cardiometabolic regulation.

Physiological process	Receptor/target	Action
Glucose homeostasis	GPR41 (FFAR3)	Activation improves insulin sensitivity and regulates energy balance through modulation of sympathetic nervous system activity
	GPR43 (FFAR2)	Enhances insulin secretion and improves glucose tolerance through incretin (GLP-1) signaling in intestinal L cells
	HDAC inhibition (butyrate)	Promotes gene expression associated with glucose metabolism and improves insulin sensitivity
Lipid metabolism	GPR43 (FFAR2)	Suppresses adipose tissue fat accumulation and promotes lipid utilization
	GPR41 (FFAR3)	Regulates lipid metabolism via effects on energy expenditure and fatty acid oxidation
	HDAC inhibition (butyrate)	Enhances mitochondrial function and fatty acid oxidation in peripheral tissues
Energy homeostasis	GPR41 (FFAR3)	Increases energy expenditure through activation of sympathetic pathways
	GPR43 (FFAR2)	Regulates adiposity by limiting fat accumulation under conditions of excess energy intake
	AMPK activation (propionate, butyrate)	Promotes energy utilization and improves metabolic efficiency
Appetite regulation	GPR43 (FFAR2)	Stimulates release of GLP-1 and peptide YY (PYY), leading to reduced appetite and food intake
	GPR41 (FFAR3)	Contributes to satiety signaling via gut–brain axis pathways
Inflammation and immune regulation	GPR43 (FFAR2)	Inhibits inflammatory signaling and promotes neutrophil recruitment and resolution of inflammation
	GPR109A	Mediates anti-inflammatory effects and promotes regulatory T-cell (Treg) differentiation
	HDAC inhibition (butyrate)	Suppresses NF-κB signaling and reduces pro-inflammatory cytokine production
Intestinal barrier integrity	GPR109A	Enhances epithelial barrier function and promotes mucosal homeostasis
	HDAC inhibition (butyrate)	Strengthens tight junctions and supports colonocyte health
Blood pressure regulation	GPR41 (FFAR3)	Modulates vascular tone and contributes to blood pressure regulation via host–microbe signaling pathways

The human gut microbiota synthesizes a variety of SCFAs. Butyrate, for instance, is produced through the anaerobic fermentation of dietary fiber by gut bacteria via the Acetyl-CoA (*ACoA*) pathway. It acts as the primary energy substrate of colonic epithelial cells. The terminal enzymatic steps of this pathway are encoded by *but* (butyryl-CoA:acetate CoA-transferase), *buk* (butyrate kinase), *bcd* (butyryl-CoA dehydrogenase), and *bcs* (butyryl-CoA synthetase). The expression of these genes reflects the fermentative capacity of the microbiome. Acetate and propionate genes are also frequently suppressed in cardiometabolic diseases. In the host, acetate acts as an energy source as well as playing a beneficial role in epithelial integrity. Its production is mainly mediated by phosphotransacetylase (*Pta*) - acetate kinase (*ackA*) pathway (*pta-ackA* pathway) (Hosmer et al., 2023). In this pathway, Phosphotransacetylase (Pta) catalyzes the conversion of Acetyl-CoA into Acetyl-phosphate. Acetate Kinase (*ackA*) then catalyzes the conversion of Acetyl-phosphate into acetate and an ATP molecule. Another pathway is mediated by the Acetyl-CoA synthetase (*acs*) gene whole role is the conversion of acetate back to Acetyl-CoA for use in the TCA cycle and other metabolic processes. Propionate, on the other hand, is formed primarily through the propanediol (*Pdiol*). Propionate exerts beneficial effects via G-protein coupled receptor 43 (GPR43)-mediated stimulation of intestinal gluconeogenesis and AMP-activated protein kinase (AMPK) signaling in the liver (Yoshida et al., 2019).Its depletion in metabolic diseases is therefore consistent with hepatic glucose regulation. However, direct causal evidence in humans linking propionate-pathway gene depletion to glucose dysregulation remains limited. Evidence from a large-scale screening of over 3,700 genomes shows that the abundance of genes associated with *ACoA*, *Pdiol* and *Suc* pathways were significantly associated with the concentrations of respective SCFAs (Kircher et al., 2022). This association supports the potential utility of the SCFA-pathway gene abundance as a biomarker of cardiometabolic. However, it is important to note that the association alone does not necessarily establish a causal or directional relationship between gene depletion and disease.

In metabolic disorders, the temporal dimension of functional losses has been resolved through longitudinal metagenomic profiling. In type 2 diabetes (T2D), for instance, metagenomic studies have consistently reported depletion of butyrate-producing taxa such as *Roseburia intestinalis* and *Faecalibacterium prausnitzii* (Qin et al., 2012). At a functional level, individuals with type 2 diabetes often show a depletion of butyrate producing taxa and therefore lower abundance of butyrate pathway genes such as *but*, *buk, bcd*, *bcs*. These functional shifts can be seen in metagenomic datasets and have been proposed as metagenomic classifiers for diabetic status (Qin et al., 2012; Vital et al., 2014b). In T1D, longitudinal analysis within the TEDDY (The Environmental Determinants of Diabetes in the Young) cohort identified reduced abundance of fermentation and SCFA biosynthesis genes as the most consistent functional alteration preceding seroconversion (Vatanen et al., 2018). In the TEDDY cohort, propanoate and butanoate metabolism were among the most strongly depleted KEGG pathways. Despite geographic and compositional variability, this signal remained comparatively stable across cohorts. A subsequent multi-omics investigation reported reduced abundance of fermentation-associated KEGG pathways, with propanoate and butanoate metabolism genes among the most strongly downregulated (Yuan et al., 2022). As with many microbiome-disease associations, the magnitude of this depletion varies across cohorts and studies differ in whether associations remain statistically significant after adjustment for diet, BMI, and medication use.

### 4.2 LPS biosynthesis and metabolic inflammation

Another recurring genetic signature in metabolic disorders is the alteration in the genes involved in host–microbial signaling and inflammation. In this regard, one of the important metabolites of gut microbiota is lipopolysaccharide (LPS) that is in return associated with risks of various metabolic disorders. LPS is a structural component of the outer membrane of Gram-negative bacteria that is assembled through a tightly regulated biosynthetic cascade encoded by the *lpx* gene family. The pathway proceeds through the sequential enzymatic activities of LpxA (UDP-N-acetylglucosamine acyltransferase), *LpxB* (lipid A disaccharide synthase), *LpxC* (UDP-3-O-acyl-GlcNAc deacetylase), *LpxD* (UDP-3-O-acyl-GlcNAc acetyltransferase), *LpxH* (UDP-2,3-diacylglucosamine hydrolase), *LpxK* (tetraacyldisaccharide 4’-kinase), *LpxL* and *LpxM* (late-stage acyltransferases) to produce Lipid A, a bioactive, pro-inflammatory moiety recognized by the host’ Toll-like Receptor 4 (TLR4) (Bertani and Ruiz, 2018; Hummels, 2025). When circulating LPS engages TLR4 on macrophages, hepatocytes, and adipocytes, it triggers MyD88-dependent activation of NF-κB. This in turn drives the transcription of TNF-α, IL-6, and IL-1β, which impairs insulin receptor substrate (IRS-1) signaling through serine phosphorylation (Kim and Sears, 2010). This molecular mechanism positions the *lpx* gene cluster as a functional bridge between gut microbial community composition and systemic metabolic inflammation.

High-fat diets and obesity may increase the proportion of LPS-containing bacteria and elevate circulating LPS, resulting in metabolic endotoxemia (Dong et al., 2022; Jawamis et al., 2025). Therefore, genes responsible for LPS enzymes (e.g. *LpxA/B/C/D/H/K/L/M*) may be predominant in individuals with metabolic inflammation and type 2 diabetes (Dong et al., 2022). These observations support the hypothesis that an increased microbial capacity for LPS biosynthesis is a recurring functional feature of metabolic disorders. However, most of the available evidence is derived from cross-sectional metagenomic studies, which identify associations rather than establish causality. For example, the metagenomic analysis of a European female cohort with T2D showed that genes involved in production of *LpxA/B/C/D/H/K/L/M* enzymes were present in patients with T2D (Dong et al., 2022). In another study, 50 postmenopausal women was analyzed for investigation of the relationship between visceral adiposity tissue (VAT) and gut bacterial microbiome (Gaber et al., 2024). After mining genome data from bacterial taxonomic units, it emerged that the expression of LPS genes (*lpxA* and *lpxB*) was higher in the group with high VAT. LPS-expressing bacteria was also abundant in the high VAT group (Gaber et al., 2024). These studies support the view that shifts in LPS-related gene abundance can contribute to pathogenesis.

An important caveat applies to this body of evidence. Most metagenomic studies infer the potential for lipopolysaccharide (LPS) production from the abundance of LPS biosynthetic genes rather than directly measuring circulating bioactive LPS. Still, quantifying plasma LPS remains technically challenging. As Munford (2016) has explained, LPS exists in structurally diverse forms, binds to lipoproteins and host proteins, and can be enzymatically inactivated. This makes it the existing endotoxin assays unreliable indicators of biologically active circulating LPS (Munford, 2016). Thus, while the enrichment of lpx genes suggests an increased microbial capacity for LPS biosynthesis, it should not be interpreted as direct evidence of elevated systemic endotoxin exposure. This does not invalidate the lpx gene-abundance findings reported above, which are a genomic rather than a circulating-protein measurement. Instead, it implies the need for the use of complementary approaches such as direct measurement of bioactive LPS, host inflammatory responses, and multi-omics validation.

### 4.3 Bile acid metabolism and host signaling

Bile acid modifying genes are also frequently disturbed in cardiometabolic disorders. Gut microbiota-mediated bile acid metabolism occupies a link between lipid homeostasis, glucose regulation, and systemic metabolic signaling. Primary bile acids like cholic acid (CA) and chenodeoxycholic acid (CDCA) are synthesized from cholesterol in the liver, then conjugated to glycine or taurine, and secreted into the duodenum (Song et al., 2024). In the distal ileum and colon, gut bacteria enzymatically transform these primary conjugates into secondary bile acids through a series of reactions whose rate-limiting steps are encoded by *bsh* (bile salt hydrolase), *hsdH* (hydroxysteroid dehydrogenase), and members of the *baiABCDEFGHI* operon responsible for 7α-dehydroxylation (Kiriyama and Nochi, 2021). Thus, gut microbiota-mediated bile-acid metabolism is consistently altered in metabolic disorders like obesity and type 2 diabetes. However, the direction and magnitude of individual gene changes may vary considerably between studies. This is likely due to differences in host diet, medication use, geographical populations, and bioinformatic pipelines. Hence, alterations in overall bile acid functional capacity may represent a more reproducible feature than changes in individual taxa or genes.

Bile acid modification represents an important layer of microbial control lipid metabolism (Bourgin et al., 2021). Indeed, several studies have documented the hypocholesterolemic effect of probiotic bacteria like *Lactobacillus* species (Liong and Shah, 2005; Nallala and Jeevaratnam, 2019; Thakkar et al., 2020). Genes encoding bile salt hydrolases (*bsh*) and hydroxysteroid dehydrogenases (*hsdH*) catalyze the deconjugation and transformation of bile acids (Poland and Flynn, 2021; Liu et al., 2025). The farnesoid X receptor (FXR) and G protein–coupled receptor (e.g. TGR5) act as bile acid sensors and therefore have a critical role in the synthesis and transport (Poland and Flynn, 2021). The bile-acid–modifying genes are widespread in gut microbes including *Bacteroides fragilis*, *Bacteroides vulgatus*, *Bifidobacterium* species, and *Lactobacillus*, suggesting that bile metabolism is a core microbial function in healthy individuals (Bourgin et al., 2021). The widespread distribution of these genes across phylogenetically distinct bacterial taxa suggests that functional redundancy within the gut microbiome may preserve bile acid metabolism despite taxonomic variation. As such, shifts in microbial composition do not necessarily translate into equivalent functional impairment. Thus, there is a need for gene-centric rather than taxonomy-centric analyses. The genetic capacity also links the microbiome to host lipid and glucose regulation, influencing pathways involved in cholesterol turnover and insulin sensitivity. The implication of the role of *bshs* is that disruptions in normal gut microbiota could impair bile metabolism and therefore result in certain diseases or conditions like lipidemia.

Metagenomic analysis show that patients with metabolic diseases have reduced abundance of bile salt hydrolase (*bsh*) genes, 7 α-dehydroxylase gene (*adh*), and 7 α-dehydroxylation (*hsdh*) gene families (Labbé et al., 2014). As a result, BSH active probiotics have been proposed and demonstrated to improve bile acid metabolism (Martoni et al., 2015). Another study has shown that increased abundance of bile acid-coenzyme A ligase (*baiB*), 3α-hydroxysteroid dehydrogenase (*baiA*), and 7α-hydroxysteroid dehydrogenase (7α-HSDH) were elevated in individuals with NAFLD (Jiao et al., 2021). The consequences of this depletion are mechanistically significant. Gut microbes are involved in the conversion of conjugated bile acids into unconjugated primary bile acids for storage in the gallbladder. Following food intake, the gallbladder contracts and releases bile into the duodenum, where these bile acids interact with the gut microbiota and modifies their signaling functions. Unconjugated bile acids can activate the farnesoid X receptor (FXR) leading to the production of FGF19 in humans. This hormone then travels to the liver, where it binds to its receptor and suppresses the liver-specific 7α hydroxylase (CYP7A1) synthesis, thereby establishing a feedback loop that regulates and reduces bile acid production (Joyce and Gahan, 2016). This means that skewed secondary bile acids profiles may be correlated with some disease-causing physiological statuses.

In nonalcoholic fatty liver disease (NAFLD), the bile acid gene signature shifts in a distinct direction. Elevated abundance of *baiB* (bile acid-CoA ligase), *baiA* (3α-hydroxysteroid dehydrogenase), and *7α-HSDH* (7α-hydroxysteroid dehydrogenase) has been reported in individuals with NAFLD (Jiao et al., 2021). This implies an overactivation of certain branches of the secondary bile acid pathway. This skewed secondary bile acid profile may modulate hepatic bile acid synthesis and glucose and lipid metabolism via signaling molecules like G protein-coupled BA receptor (GPBAR1 or TGR5) and farnesoid X receptor (FXR) (Jiao et al., 2021). Interestingly, Jiao et al. (2021) did not observe significant changes in the abundance of microbes involved in bile acid metabolism. Also noteworthy is an important limitation of the study in that effects of demographic and environmental variables on obesity and diabetes on the microbiome was not measured. So, it is likely that extraneous demographic and environmental factors had profound impacts on gut microbiota profiles. The alteration in the bile acid synthesis feedback may amplify inflammatory NF-κB activity as well as suppress bile acid synthesis. Thus, BSH-active probiotics have been proposed and experimentally demonstrated to normalize bile acid metabolism by restoring *bsh* gene activity (Huang et al., 2020).

An important caveat must be considered when the interpreting alterations in bile acid-modifying genes. Even though the changes in *bsh*, *bai*, and *hsdH* abundance point to altered microbial capacity for bile acid transformation, the physiological consequences are highly context dependent. This is due to the fact that different bile acid species exert distinct and sometimes opposing effects on host signaling pathways. For example, FXR activation is generally regarded to be metabolically beneficial. However, the metabolic outcome depends on the specific bile acid ligand and the tissue in which FXR is activated (Kemper, 2011). Intestinal FXR antagonism by microbiota-derived bile acids such as tauro-β-muricholic acid has been shown to improve obesity and glucose homeostasis, whereas systemic FXR activation may produce different metabolic effects (Sun et al., 2018; Ding et al., 2024). Therefore, changes in *bsh*, *bai*, or *hsdH* abundance should not be interpreted as uniformly protective or detrimental without concurrent characterization of the bile acid pool, receptor activation, and tissue-specific signaling. This complexity highlights an important limitation of inferring metabolic outcomes solely from metagenomic measurements of bile acid-related genes. It also emphasizes the need for integrated metagenomic, metabolomic, and functional studies.

### 4.4 Amino acid and BCAA metabolism

In addition to bile acid metabolism, there is growing evidence that microbial amino-acid metabolism genes are associated with insulin resistance and elevated circulating branched-chain amino acids (BCAAs) in insulin resistance and cardiometabolic disease. BCAAs are a source of nutrients where they form building blocks for proteosynthesis or as a substrate for glucogenic energy generation (Gojda and Cahova, 2021). They also serve as signaling molecules via the mTOR activation pathway (Gojda and Cahova, 2021). BCAAs (e.g. leucine, isoleucine, and valine) act as potent activators of rapamycin complex 1 (mTORC1) and its downstream effectors, including ribosomal protein S6 kinase 1 (S6K1) and 4E-BP1 (Ishiguro et al., 2006; Mei et al., 2025). Even though BCAA concentrations support anabolic signaling, chronically elevated circulating BCAAs are strongly associated with insulin resistance, impaired glucose tolerance, and increased risk of T2D. This is consistent with the observation that mTORC1/S6K1 hyperactivation directly phosphorylates IRS-1 at inhibitory serine residues, thereby uncoupling the insulin receptor from its downstream PI3K/Akt effectors (Veilleux et al., 2010). Although elevated circulating levels of BCAAs are among the most reproducible metabolic biomarkers of insulin resistance, it is unclear whether they act as causal mediators or simply reflect impaired metabolic health (Lynch and Adams, 2014). One hypothesis of the link between BCAAs levels and T2DM is that mTORC1 activation plays a role in disrupting insulin signaling. Yet some studies also indicate that reduced host BCAA catabolism and mitochondrial dysfunction may contribute substantially to BCAA accumulation. This biological duality is what invited Li and colleagues call this an “angle or demon” phenomenon (Li et al., 2026). Consequently, elevated BCAAs are increasingly viewed as both potential drivers and biomarkers of metabolic dysfunction rather than unequivocal causal agents.

The gut microbiome’s contribution to circulating BCAA levels is now understood to be both quantitatively important and gene-dependent. Multiple studies indicate that shifts in microbial BCAA gene content and transport systems correlate with serum metabolite signatures linked to impaired glucose metabolism and other metabolic outcomes. For example, in a Danish cohort comprising 277 non-diabetic individuals, insulin resistance was found to be strongly correlated with increased levels of BCAAs and the genes encoding for them (Pedersen et al., 2016). The increase in microbial BCAAs was largely driven by *Prevotella copri* and *Bacteroides vulgatus*. Pedersen et al. provided one of the strongest pieces of evidence linking microbial BCAA biosynthetic capacity to host insulin resistance by combining metagenomics, metabolomics, and fecal transplantation experiments. They moved beyond purely associative observations. However, the contribution of *P. copri* appears to be context dependent. This is due to the fact that this species has been associated with both beneficial and detrimental metabolic outcomes depending on strain diversity, dietary background, and host population (Abdelsalam et al., 2023; Wang et al., 2026). In another study involving a cohort of Chinese individuals, gut microbial signatures in obese individuals were linked to changes in circulating metabolites with elevated pathways for BCAAs (Liu et al., 2017). Similarly, a recent study involving a metagenomic analysis of gut microbiome has shown that pathways linked to BCAA and bile acid metabolism are elevated in individuals with diabetes (Morsy et al., 2025). These studies position microbial BCAA and amino acid metabolism genes as important mechanistic links in the chain connecting gut dysbiosis to systemic metabolic dysfunction. The consistency between geographically diverse cohorts suggests that alterations in microbial BCAA metabolism may represent a conserved functional feature across geographically distinct populations. However, differences in dietary patterns, obesity severity, medication use, and microbiome composition may still make direct comparisons challenging. It is also important to note that most human studies remain observational. This makes it difficult to determine the relative contributions of microbial BCAA production, host amino acid catabolism, and dietary protein intake to circulating BCAA concentrations.

The direction of causality in the BCAA-insulin resistance relationship remains contested in the human genetics literature. So, since the studies summarized above entail cross-sectional metagenomic associations, they cannot resolve this question. Mendelian randomization (MR) has been explored as a solution since it exploits genetic variants as instruments to reduce confounding and reverse causation. In this field, Lotta and colleagues found evidence consistent with a causal role of impaired BCAA catabolism in the development of type 2 diabetes (Lotta et al., 2016). However, subsequent Mendelian randomization analyses using different genetic instruments reached the opposite conclusion (Mahendran et al., 2017). These MR studies found genetic support for insulin resistance as a cause of elevated circulating BCAAs rather than the reverse (Mahendran et al., 2017; Wang et al., 2017).

### 4.5 TMA/TMAO and cardiovascular risk

Among the gut microbiome-derived metabolic axes associated with cardiometabolic disease, the trimethylamine/trimethylamine N-oxide (TMA/TMAO) pathway has attracted particular attention in cardiovascular research. This is due to the specificity of its enzymes, the mechanistic clarity of its pro-atherogenic effects, and the tractability of its key genes as therapeutic targets. TMA is an inorganic compound that is generated by gut microbiota from dietary quaternary amines like choline, phosphatidylcholine, L-carnitine, and betaine, which are abundant in red meat, eggs, fish, and poultry (Gatarek and Kaluzna-Czaplinska, 2021). They are formed through the action of distinct microbial enzyme complexes (TMA lyases) whose encoding genes have emerged to be an important functional fingerprint of cardiovascular risk (Tang et al., 2017). TMAO, on the other hand, is a hepatic oxidation product of TMA. TMA/TMAO pathway is one of the best-characterized examples of host–microbiome co-metabolism because of its clearly defined microbial enzymes and host metabolic processing. However, there is still no scientific consensus on the extent to which circulating TMAO acts as a causal mediator rather than a biomarker of cardiometabolic dysfunction (Papandreou et al., 2020; Canyelles et al., 2023).

The primary TMA-producing enzyme systems are encoded by three well-characterized gene clusters. The *cutC/D* operon encodes choline TMA-lyase (*CutC*) and its related activating enzyme (*CutD*) (Craciun and Balskus, 2012). *CutC* is a glycyl radical enzyme that abstracts a hydrogen atom from choline at the C1 position via a thiyl radical intermediate, resulting in anaerobic TMA release. On the other hand, *CutD* functions as a radical S-adenosylmethionine activase required for *CutC* activation (Craciun and Balskus, 2012). The complementary *cntA/B* system encodes a two-component Rieske-type oxygenase/reductase complex that uses L-carnitine as substrate, oxidizing it at the C-N bond to yield TMA and malic semialdehyde (Massmig et al., 2020). *cntA/B* is prominently expressed in a variety of bacterial strains including *Acinetobacter calcoaceticus* and *Serratia marcescens* (Cai et al., 2022). A third gene system, *yeaW/X*, shares close sequence identity with *cntA*/B and exhibits broader substrate promiscuity by utilizing choline, γ-butyrobetaine, and betaine in addition to carnitine (Cai et al., 2022). A bioinformatics screening of over 67,000 microbial genomes has identified more than 1,100 candidate *cutC*-encoding and over 6,700 *cntA*-encoding organisms, distributed across Firmicutes, Proteobacteria, and Actinobacteria (Rath et al., 2017). The phylogenetic distribution of these genes suggests that TMA production is a conserved microbial function. So, cardiovascular risk is likely influenced more by the collective abundance and activity of TMA-producing pathways than by the presence of individual bacterial taxa. This observation further supports the value of functional metagenomics over taxonomic profiling when evaluating microbiome-associated cardiovascular risk.

**Figure 2:**
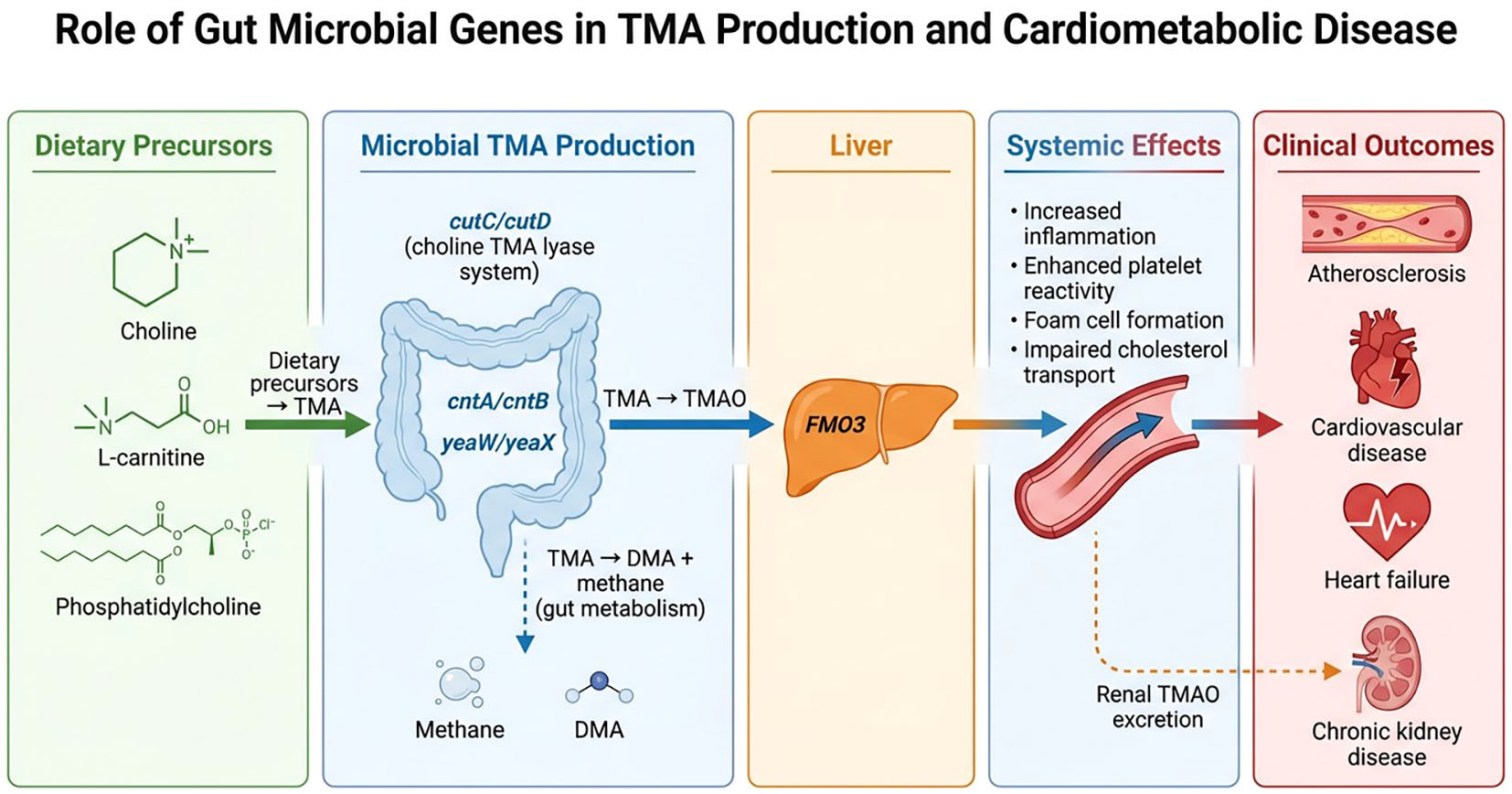
Microbial functional pathways involved in trimethylamine (TMA) production and their contribution to cardiometabolic disease. Figure created with the assistance of FigureLabs and refined by the author for scientific accuracy.

Cardiovascular risk may be established through a screening of TMA/TMAO associated genes. TMA absorbed from the gut is transported via the portal circulation to the liver, where flavin-containing monooxygenase 3 (FMO3) oxidizes it to TMAO (**Figure 2**). As illustrated in **Figure 2**, elevated circulating TMAO promotes atherosclerosis through several converging mechanisms: 1) It stimulates macrophage scavenger receptor expression, 2) It enhances foam cell formation; and 3) It inhibits reverse cholesterol transport by suppressing hepatic bile acid synthetic enzymes (e.g. *CYP7A1* and *CYP27A1*); 4) It activates the MAPK/NF-κB inflammatory cascade in endothelial cells; and 5) It potentiates platelet hyperreactivity and thrombosis through *CutC/D*-dependent mechanisms (Zhu et al., 2016; Roberts et al., 2018; Wang et al., 2021). These mechanisms provide a strong biological plausibility for a contributory role of the TMA/TMAO pathway in cardiovascular disease. However, the relative contribution of each mechanism to human disease progression remains incompletely understood. This is mainly due to the complex interactions between TMAO, renal function, dietary intake, and host metabolism. Integrative metagenomics of atherosclerotic patients has identified *Lachnoclostridium*, the most abundant *cutC*-containing genus in healthy individuals, as significantly overrepresented in atherosclerotic disease (Cai et al., 2022). Specifically, *L. saccharolyticum* WM1 demonstrated near-complete choline-to-TMA conversion efficiency in gnotobiotic ApoE^−/−^ mouse models (Cai et al., 2022). These mechanistic and clinical convergences suggests that the *cutC*/*cutD*/*cntA* gene axis as both a biomarker of cardiovascular risk and a therapeutic target amenable to substrate competition, non-lethal enzymatic inhibition, and microbiome-targeted dietary intervention. TMA production may also be overexpressed in some metabolic diseases including obesity (Schugar et al., 2022). Nevertheless, translating these findings requires caution since microbial metabolism and dietary exposures often differ between experimental models and clinical populations.

Dietary nutrients such as choline, L-carnitine, and phosphatidylcholine are metabolized by the gut microbiome into trimethylamine (TMA) through the activity of microbial enzymes encoded by gene clusters including cutC/cutD, cntA/cntB, and yeaW/yeaX. TMA is subsequently absorbed into the circulation and oxidized in the liver by flavin-containing monooxygenase 3 (FMO3) to form trimethylamine N-oxide (TMAO). Elevated circulating TMAO levels have been associated with multiple pathophysiological processes, including enhanced platelet reactivity, increased inflammatory signaling, foam cell formation, and impaired reverse cholesterol transport. These functional effects contribute to the development and progression of cardiometabolic conditions such as atherosclerosis, cardiovascular disease, heart failure, and chronic kidney disease. A fraction of TMA may undergo further microbial metabolism to dimethylamine (DMA) and methane, while TMAO is primarily cleared through renal excretion.

As described above there is a mechanistic clarity on the cutC/cntA-TMAO-atherosclerosis pathway. However, the clinical evidence linking circulating TMAO to cardiovascular outcomes in humans is considerably less consistent. A 2024 review of data on TMAO as a cardiovascular biomarker concluded that its performance across studies has been conflicting (Jaworska et al., 2024). The authors attributed this observation to confounding factors that affect TMAO plasma levels such as kidney function, diet, and demographic variability (Jaworska et al., 2024). Elsewhere, a meta-analysis focused specifically on myocardial infarction patients did find significant positive associations between TMAO and major adverse cardiovascular events. However, no significant association was established for stroke, coronary complexity, or multivessel disease, and explicitly noted that the definition of “elevated” TMAO varied substantially across studies (Li et al., 2024b). This outcome-dependent pattern suggests TMAO’s clinical utility may be more outcome-specific than the general mechanistic narrative implies.

### 4.6 Carbohydrate-active enzymes and energy harvest

The capacity of the gut microbiome to extract calories from otherwise indigestible dietary carbohydrates is governed by a vast and structurally diverse arsenal of carbohydrate-active enzymes (CAZymes). These are a functional gene category whose dysregulation in cardiometabolic disease reflects a compositional imbalance and a fundamental reprogramming of the microbiome’s energy-harvesting potential. CAZymes encompass glycoside hydrolases (GHs), polysaccharide lyases (PLs), carbohydrate esterases (CEs), and glycosyltransferases (GTs) (Barbe et al., 2026). The GH families constituting the most functionally characterized and metabolically consequential group in obesity and metabolic syndrome. CAZymes are responsible for starch and glycogen degradation and are frequently expressed by Firmicutes and Bacteroidetes. Most importantly, individuals with different CAZyme profiles within their gut microbiomes may have different metabolic capabilities in terms of degradation and absorption of carbohydrates on the intestinal epithelium (Bhattacharya et al., 2015). In other words, these enzyme families determine the efficiency of fiber fermentation and the completeness of SCFAs fermentation with important implications for metabolic outcomes. While CAZyme profiles might provide insights into the metabolic potential of the gut microbiome, there is an important caveat. Functional redundancy among microbial communities cannot be ruled out and this means that similar carbohydrate-degrading capabilities may be maintained despite substantial taxonomic variation. As a result, alterations in CAZyme abundance are likely to be more reproducible across populations than changes in individual bacterial species.

Genetic signatures have been reported in metabolic syndromes associated with gut microbiome dynamics. A seminal observation that the obese microbiome possesses an increased capacity to harvest energy from the diet was established through metagenomic and biochemical analysis (Turnbaugh et al., 2006). The study demonstrated that the Firmicutes-enriched and Bacteroidetes-depleted microbial communities encoded a functionally enhanced carbohydrate metabolism repertoire. This landmark study established the concept that the gut microbiome can influence host energy harvest. However, subsequent studies have suggested that the relationship between microbial energy extraction and obesity is more complex than initially proposed (Magne et al., 2020). Specifically, the widely cited increase in the Firmicutes-to-Bacteroidetes ratio has not been consistently replicated across independent human cohorts (Walters et al., 2014; Sze and Schloss, 2016; Magne et al., 2020). Sze and Schloss (2016) reprocessed raw sequencing data from ten independent human studies using a single, uniform pipeline. They found that the Firmicutes-to-Bacteroidetes ratio was not significantly associated with obesity in any of the ten studies individually. This means that functional capacity may represent a more robust marker of metabolic dysfunction than broad taxonomic composition. Elsewhere, a global profiling of CAZyme families across 448 individuals from diverse geographic backgrounds identified a group of GH families showing positive correlation with BMI (Bhattacharya et al., 2015). These genes were specifically enriched in the Firmicutes phylum and were organized into distinct individuals sharing similar CAZyme repertoire profiles with corresponding dietary and metabolic signatures known as “CAZotypes.” However, whether these functional signatures remain stable over time or are primarily shaped by habitual diet and other environmental factors remains inconclusive. Other studies have reported that genes linked to carbohydrate-active enzymes (CAZyme) (e.g., GH13, GH43, GH3 families) may be elevated in individuals with obesity due to increased capacity for caloric extraction (Diener et al., 2021; Blecksmith et al., 2025). These findings support the hypothesis that enhanced microbial carbohydrate-degrading capacity contributes to increased energy availability. But the inherent limitation of metagenomic studies still persists. These studies quantify the abundance of CAZyme genes rather than their enzymatic activity or substrate utilization. Therefore, they cannot be used to determine whether increased genetic potential directly translates into greater caloric extraction *in vivo*. This limitation calls for an integration of metagenomics with metatranscriptomics, metaproteomics, and isotope-based metabolic flux analyses to validate these functional predictions. Further observations point to the possibility that taxa involved in butyrate synthesis may be diminished in individuals with T2D and obesity (Arora and Tremaroli, 2021). This means a corresponding depletion of butyrate synthesis genes (e.g. *but, buk, bcd, bcs*). Oxidative stress response and production of pro-inflammatory cytokines IL-6 and TNF α may also be enriched in T2D (Lazar et al., 2019). These observations suggest that alterations in CAZyme repertoires extend beyond nutrient acquisition to influence microbial metabolic outputs and host inflammatory responses. Still, the current evidence remains largely associative. It is therefore important to note that microbial carbohydrate metabolism to obesity may be influenced by host genetics, dietary composition, and lifestyle factors.

## 5 Translational potential and future directions

Advances in multi-omics research have equipped us with knowledge of functional microbial genes of the gut microbiome. Now, the next phase will involve the translation of this knowledge into genuine translational applications. The functional genetic signatures highlighted in this review constitute plausible targets for a range of potential therapeutic and diagnostic strategies. However, a note of caution is necessary throughout this section. None of the approaches discussed below has reached prospective clinical validation specifically for a cardiometabolic indication. Indeed, most of the supporting evidence below is preclinical or drawn from small, short-duration human studies.

### 5.1 Functional gene panels as cardiometabolic biomarkers

There is an increasing need to identify robust biomarkers that can inform the diagnosis and prognosis of cardiometabolic diseases. However, translating high-throughput microbiome data into clinically useful biomarkers remains a considerable challenge. To be effective, microbiome-derived biomarkers must demonstrate adequate sensitivity, specificity, and reproducibility across diverse populations. In this context, functional genetic signatures offer a promising alternative to taxonomic markers, as they reflect conserved metabolic capabilities rather than variable microbial composition. In cardiometabolic disorders, several functional gene patterns have been consistently observed. These include the depletion of short-chain fatty acid (SCFA) biosynthesis genes, reduced abundance of bile salt hydrolase genes, and enrichment of lipopolysaccharide (LPS) biosynthesis pathways. For example, the downregulation of genes responsible for the synthesis of acetate (*ackA*, *pta*) and butyrate (*buk*, *but*) has been identified as a biomarker for acute pancreatitis (Deng et al., 2025). Such alterations are detectable in metagenomic datasets and may serve as indicators of metabolic imbalance. Some of these functional shifts appear prior to overt clinical manifestations, suggesting potential utility in early detection and risk stratification. Still, no functional gene panel discussed in this review has been validated as a diagnostic or risk-stratification tool in a cardiometabolic-disease-specific clinical trial.

**Figure 3:**
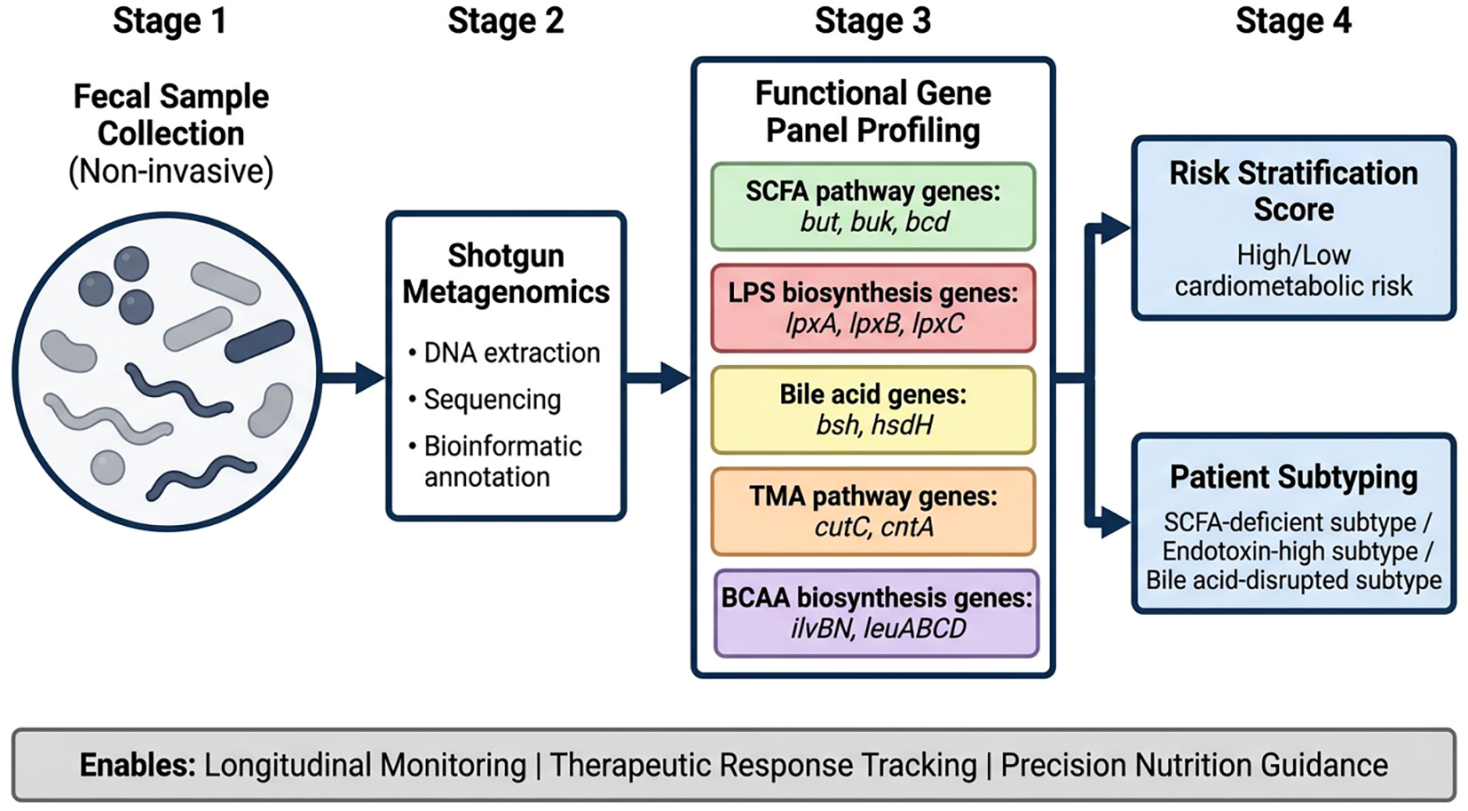
A proposed framework for the use of functional genetic signatures as biomarkers for early detection of cardiometabolic diseases.

The development of gene-based biomarker panels represents a practical step toward clinical application. These panels could be designed to capture key functional pathways and implemented using targeted sequencing or quantitative approaches. Based on these genetic signatures, clinicians may stratify individuals based on the risk score or even use the data for patient subtyping (**Figure 3**). **Figure 3** outlines the proposed workflow from metagenomic sequencing through functional gene panel construction to risk stratification and patient subtyping. The figure represents a conceptual framework rather than a validated clinical pipeline. As previously noted above, no functional gene panel discussed in this review has yet been tested prospectively in a cardiometabolic-disease-specific cohort. So, the risk-stratification and patient-subtyping outputs shown here remain proposed applications rather than demonstrated capabilities. Importantly, several challenges persist. Inter-individual variability, differences in sequencing platforms, and the need for standardized analytical frameworks may limit reproducibility. Furthermore, functional signatures identified in gut samples must be linked to measurable changes in peripheral tissues or biofluids to enhance clinical feasibility. Integrative approaches that combine microbiome data with metabolomic and clinical parameters may help bridge this gap and improve biomarker performance.

### 5.2 Fecal microbiota transplantation

One of the potential applications involves the use of fecal microbiota transplantation (FMT). Originally validated for recurrent *Clostridioides difficile* infection (rCDI), FMT achieved landmark regulatory recognition in 2022 and 2023 when the US FDA approved it for fecal microbiota, live-jslm and fecal microbiota spores, and live-brpk (Miller et al., 2026). For rCDI, FMT has been reported to achieve cure rates of >90% (Miller et al., 2026). Beyond infections treatment, FMT has been reported to improve certain pathways in the recipients. For example, in a mouse model, colitis individuals who received FMT showed increased abundance of *Bacteroides thetaiotaomicron* and *Faecalibacterium prausnitzii* as well as functional pathways for lecithin in the glycerophospholipid metabolism (Xu et al., 2024). However, these findings come from a mouse model of colitis rather than a cardiometabolic disease or human study. As such, the work should be viewed as illustrative. For cardiometabolic diseases, FMT has been reported to reverse insulin resistance in patients with type 2 diabetes. In a Chinese cohort, individuals who received FMT and metformin were reported to have an improvement in clinical indicators and BMI in T2DM (Wu et al., 2023). However, this prospective study involved a small cohort of 31 individuals. It also involved a short study period of four weeks, which made it difficult to understand long-term clinical efficacy. A meta-analysis of FMT interventions for weight and glycemic control has shown that FMT could lower BMI, weight, and HbA1c levels (Hu et al., 2023). Another meta-analysis of 10 randomized control trials established that FMT could have a beneficial effect on modulation of lipid metabolism in patients with metabolic syndrome (Wang et al., 2024). Still, FMT still faces numerous challenges and its utilization in clinical trials remain limited.

Besides FMT, another potential application relates to the use of live biotherapeutic products. In a randomized double-blind, placebo-controlled pilot trial of 32 overweight or obese participants, *Akkermansia muciniphila* supplementation was found associated with improved insulin sensitivity, reduced insulinemia, and total cholesterol (Depommier et al., 2019). The study points to the rationale for postbiotic development. However, it must be viewed as a reasonable proof of concept since the trial was small, and short in duration. Also, insulin sensitivity and lipid markers were used as surrogate endpoints rather than hard cardiometabolic outcomes such as incident diabetes or cardiovascular events. Hence, it should be read as an early-phase signal rather than established evidence of clinical benefit.

### 5.3 *In situ* engineering

Engineering microbial strains for improved gut homeostasis is an actively developing approach but it remains early-stage and preclinical for cardiometabolic applications. Understanding which functional genes are enriched or depleted in disease states provides a starting point for developing strains that correct these imbalances. For example, synthetic biology tools have been used to develop strains that can synthesize probiotic metabolites like butyrates within the gut. This was the strategy used by Wu and colleagues. In their work, *Saccharomyces cerevisiae* was engineered to tolerate higher butyrate production by integrating pathways like 3-hydroxybutyryl-CoA dehydrogenase, 3-hydroxyacyl-CoA dehydrogenase, enoyl-CoA hydratase, enoyl-CoA hydratase, trans-2-enoyl-CoA reductase, crotonyl-CoA reductase, and acetate CoA/acetoacetate CoA-transferase (Wu et al., 2024). Individuals who received the engineered strain were found to have an improved abundance of beneficial microbes like *Bifidobacterium* and *Lactobacillus* as well as decreased disease indices (Wu et al., 2024). Similarly, administration of engineered *Escherichia coli* Nissle 1917 (EcN) in colitis animal models has been shown to restore the expression of miRNAs for improved microbial homeostasis, reduced gut inflammation, and improved anti-inflammatory effect (Rodríguez-Nogales et al., 2018). However, evidence of the utilization of these applications for cardiometabolic diseases in humans remain limited.

*In situ* genetic engineering of resident microbiota continues to attract interest in clinical and scientific research. A landmark 2024 study demonstrated that phage-derived particles loaded with a base editor achieved a median editing efficiency of 93% in *E. coli* colonizing the mouse gut (Brödel et al., 2024). In this study, edited bacteria could maintain viability for at least 42 days following a single dose. The base-editing cargo introduces precise single-nucleotide substitutions while minimizing collateral damage to the bacterial genome. Additionally, Ye et al. have validated a toolkit that can permit targeted gene knockouts and insertions in multiple human gut *Bacteroides* species (Yeh et al., 2024). Recently, Gelsinger and colleagues have introduced MetaEdit, a technique that allows species-specific editing of gut microbiota using mobile CRISPR-associated transposases in living animals at high specificity (Gelsinger et al., 2025). These are notable proof-of-principle demonstrations of the underlying editing technology. However, most of the existing studies have been conducted in animal models. This means that safe delivery in cardiometabolic diseases in humans remain limited. Still, the advances mean that individualized therapy is a path with translational potential for future development.

### 5.4 Precision phage therapy

Another potential application comes from the use of precision bacteriophage therapy. Bacteriophages are viruses that infect bacteria. They can be used in microbiome remodeling because rather than adding beneficial organisms, they selectively deplete pathobionts carrying harmful functional gene programs. This precision is particularly compelling given the gene-level dysbiosis characterized throughout this review. Phages that target *E. coli* strains carrying disadvantageous operons like lpxA/B LPS gene clusters could, in principle, reduce the inflammatory Proteobacterial populations that are characteristic of cardiometabolic conditions. Already, personalized bacteriophage therapy has demonstrated proof-of-concept in a retrospective analysis of 100 consecutive cases treated by a Belgian consortium across 35 hospitals in 12 countries (Pirnay et al., 2024). Positive clinical outcomes were achieved in 77% of cases with bacteriophage-antibiotic synergy being identified as a significant correlate of bacterial eradication. However, these cases were treated for diverse, mostly drug-resistant bacterial infections rather than for cardiometabolic dysbiosis. Also, the cohort lacked a control arm. Also, the use of phages to deplete cardiometabolic-disease-associated pathobionts as proposed above remains a theoretical extension that has not itself been tested. Moreover, the applications remain constrained by scientific and regulatory challenges (Moon et al., 2025).

### 5.5 Pharmacomicrobiomics

Lastly, pharmacomicrobiomics presents a potential area of precision translational medicine. Bolte et al. define pharmacomicrobiomics as the study of how inter-individual microbiome variation shapes drug pharmacokinetics and pharmacodynamics (Bolte et al., 2025). As described previously in this review, gut microbes have intense enzyme activities. These enzymes may inevitably modulate drug metabolism through direct and indirect actions. Gut microbiota-drug interactions are likely to affect patient outcomes. For example, the administration of antibiotics is well known to cause dysbiosis, which in turn increase the risk of various bacterial and fungal infections (Francino, 2015). Other drugs prescribed for treating stomach acid reflux, heartburn, type 2 diabetes, and heart diseases have also been implicated in causing changes in the gut microbiota (Dikeocha et al., 2022). Besides, there exists a risk of microbiota biotransformation of some drugs and causing some toxic metabolites. For example, formate C-acetyltransferase produced by Bifidobacterium has been reported to inhibit the action of the enzyme involved in acetaminophen metabolism, resulting in a risk of liver failure and hepatocyte oxidative damage (Li et al., 2024a). In cancer immunotherapy, the association between gut microbiome composition and immune checkpoint blockade (ICB) response has been robustly established (Gunjur et al., 2024). Strain-resolved metagenomic analyses revealed that strain-level functional signatures, rather than species-level taxonomic markers, best predicted ICB response (Gunjur et al., 2024). The implication of this findings is that therapeutics must take gut microbiome into consideration. In other words, a precision workflow is desirable where patients are metagenomically profiled prior to therapy initiation. Such a strategy would involve a comprehensive screening of functional genetic signatures for subsequent analysis of potential drug interactions.

## 6 Conclusion and future directions

Advances in metagenomics have improved our understanding of the gut microbiome. The gut microbiome can be viewed as a functional ecosystem that contains a large repertoire of microbial genes. These genes influence many aspects of host physiology. Along the gut-liver axis, microbial bile acid transformation (e.g. *bsh*, *bai*, *hsdH*) and TMA production (*cutC*/*cutD*, *cntA*/*cntB*) converge on hepatic receptor signaling and FMO3-mediated oxidation, respectively. Hence, a link exists between microbial gene content and hepatic lipid handling and metabolic dysfunction-associated liver disease. For the gut-vascular axis, depletion of SCFA-producing genes and enrichment of LPS biosynthesis genes (*lpxA-M*) are consistently associated with impaired vascular tone, endothelial inflammation, and atherosclerotic risk. Along the gut-immune-metabolic axis, the barrier-maintenance and immune-tolerance genes discussed earlier in this review intersect with BCAA and SCFA metabolism to link microbial function to systemic insulin resistance and low-grade inflammation. As highlighted in this review, many studies report depletion of beneficial metabolic pathways. Examples include reduced short-chain fatty acid production and the enrichment of pro-inflammatory or potentially harmful pathways. Additionally, cardiometabolic diseases have been linked to alterations in lipopolysaccharide biosynthesis, amino acid and BCAA metabolism, Trimethylamine oxide production, and the actions of CAZymes. Metagenomic studies consistently report either the increased or reduced abundance of functional genes associated with these functions. Such findings suggest that microbial functional potential is an important determinant of host health.

Future research should continue to integrate metagenomics with other omics approaches such as metabolomics and transcriptomics. These strategies will help to clarify how microbial genes translate into biological activity in the host. Identifying reliable microbial functional signatures may support the development of microbiome-based diagnostics and therapeutic strategies aimed at restoring microbial balance and improving human health. In future, researchers must explore deeper utilization of functional genetic signatures for precision medicine and other applications. First, the future studies must move beyond single-target, single-strain interventions towards a microbial ecology approach that focuses on complete and functionally restorative strategies. This will help to address the multi-layered gene program dysregulation that is characteristic of complex diseases. Second, the translational success depends on the convergence of *in situ* CRISPR editing, synthetic ecology design, phage-mediated selective depletion, and pharmacomicrobiome-stratified drug development. Additionally, AI-integrated multi-omics diagnostics will play a greater role in the future whereby each clinical decision will be informed by the functional gene architecture catalogued for each patient. This means that the gut microbiome will be treated as a druggable, diagnostically informative, and profoundly personalized dimension of precision medicine. Lastly, we must look beyond scientific research and deal with regulatory issues that might hinder the adoption of these technologies. This calls for regulatory innovation to deal with challenges of safety, equity, and data security.
